# Expert consensus on the management of adverse events and prescribing practices associated with the treatment of patients taking pirfenidone for idiopathic pulmonary fibrosis: a Delphi consensus study

**DOI:** 10.1186/s12890-020-01209-4

**Published:** 2020-07-14

**Authors:** Franck F. Rahaghi, Zeenat Safdar, Anne Whitney Brown, Joao A. de Andrade, Kevin R. Flaherty, Robert J. Kaner, Christopher S. King, Maria L. Padilla, Imre Noth, Mary Beth Scholand, Adrian Shifren, Steven D. Nathan

**Affiliations:** 1 Department of Pulmonary and Critical Care Medicine, Cleveland Clinic Florida, 2950 Cleveland Clinic Blvd, Weston, FL 33331 USA; 2 Houston Methodist Lung Center, Houston, TX USA; 3 Inova Fairfax Hospital, Falls Church, VA USA; 4 Birmingham VA Medical Center, Birmingham, AL USA; 5 Michigan Medicine Pulmonary Clinic, Ann Arbor, MI USA; 6 Weill Cornell Medicine Pulmonary & Critical Care Medicine, New York, NY USA; 7 The Mount Sinai Hospital, New York, NY USA; 8 The University of Chicago Medicine, Chicago, IL USA; 9 University of Utah School of Medicine, Salt Lake City, UT USA; 10 Washington University School of Medicine, St. Louis, MO USA

**Keywords:** Adverse events, Idiopathic pulmonary fibrosis, Management, Pirfenidone, Expert consensus

## Abstract

**Background:**

In patients with idiopathic pulmonary fibrosis (IPF) treated with pirfenidone (Esbriet®, Genentech USA, Inc. South San Francisco, CA.), effectively managing treatment-related adverse events (AEs) may improve adherence. Due to a lack of clinical evidence and expertise, managing these AEs can be challenging for patients and physicians alike. In the absence of evidence, consensus recommendations from physicians experienced in using pirfenidone to treat IPF are beneficial.

**Methods:**

Using a modified Delphi process, expert recommendations were developed by a panel of physicians experienced with using pirfenidone for IPF. Over three iterations, panelists developed and refined a series of statements on the use of pirfenidone in IPF. Their agreement on each statement was ranked using a Likert scale.

**Results:**

A panel of 12 physicians participated and developed a total of 286 statements on dosing and administration, special populations, drug-drug interactions, laboratory analysis, warnings and precautions, and AE management. Expert recommendations were achieved with regard to slower initial titrations and slower titrations for AEs, dosing with meal(s) or substantial meals, and adding other prescribed pharmacological agents for AEs.

**Conclusion:**

Until there is further clinical evidence, the resulting consensus recommendations are intended to provide direction on the practical management of IPF with pirfenidone, by encompassing a broad experience from the real world to complement data gleaned from clinical trials.

## Background

Idiopathic pulmonary fibrosis (IPF) is a rare disease characterized by a variable decline in lung function, due to progressive scarring of lung tissue [1]. The disease occurs primarily in middle-aged to older individuals [2, 3]. The incidence and prevalence rates of IPF have been rising, due to the aging population of the United States (US) [2, 3]. Globally, mortality with IPF appears to be increasing as well, however, variations exist in mortality trends and rates between different countries [4, 5]. While the cause of IPF is currently unknown, it is hypothesized that aging and genetic factors play a role in disease development [2, 3]. Effective treatment options for IPF are limited, with only two anti-fibrotic medications currently approved to treat the disease in the US [6].

Pirfenidone (Esbriet®, Genentech USA, Inc. South San Francisco, CA.) is an orally administered anti-fibrotic indicated for the treatment of IPF [7]. It was approved in the United States in 2014 based on data from the Phase III ASCEND trial, supported by two other Phase III trials, CAPACITY 1 and 2 [8–10]. Like other anti-fibrotics, pirfenidone may cause adverse events (AEs) ranging from mild GI (gastrointestinal) side effects to severe drug reactions. Discontinuation rates with pirfenidone as a result of AEs are as high as 30% [11, 12]. Furthermore, recent data from a pooled meta-analysis of Phase II and Phase III studies show that patients who remain on pirfenidone for 120 weeks or more have a clinically significant reduction in all mortality outcomes examined, thus suggesting the key to success with pirfenidone is to get the patients on treatment and maintain them on their treatment [13]. Therefore, when using anti-fibrotic medications such as pirfenidone to manage patients with IPF, education to define treatment expectations is integral to enable the prevention and effective management of AEs. This is also essential to facilitate and improve adherence to therapy [14]. Due to limited clinical experience and a narrow base of published recommendations, mitigating and preventing AEs with pirfenidone is challenging, and best practice strategies often go undefined. Pivotal trial data and prescribing information are a useful source of efficacy and safety data. Yet, these sources do not incorporate real-world or experiential learning with regard to pirfenidone and may neglect detailed information on AE management as well as the nuances of dose titration. Recent Delphi recommendations have been published for other therapies and anti-fibrotics that are used to treat IPF [15–17]. While there are a few published resources with expert opinion and real-world evidence on the topic of managing pirfenidone-associated AEs, these recommendations may be incomplete and can be further developed to provide clarity. Both Costabel et al. and Lancaster et al. previously reported AE management of pirfenidone, however, these results grouped recommendations for a broad range of AEs and do not contain specific directions for the most common AEs reported with pirfenidone [18, 19].

Despite a scarcity of clinical and evidence-based data or guidance, it is critical for physicians treating IPF to adequately address pirfenidone-related AEs and manage other specific circumstances. The expert advice from physicians experienced in the treatment of IPF with pirfenidone may benefit other physicians and their patients. The Delphi method is a proven methodology for gathering and compiling consensus advice when evidence is scarce. This Delphi study thus intends to provide consensus recommendations for the management of IPF with pirfenidone based on the current practice in the US. The expert panel in this study comprised of US-based physicians who routinely manage treatment-related AEs associated with pirfenidone.

## Methods

Sponsored and funded via Genetech (see full funding disclosure), a modified Delphi process was used to develop the consensus statements contained within this report. The Delphi process was originally described by Delbecq and colleagues, and is a proven methodology to gain consensus advice on a topic of interest [20–24]. The first and the senior author (FFR), and second authors (ZS) and (SDN) served as study moderators and recruiters of the Delphi panelists. The target Delphi panel size was 15–20 members, where US investigators from the ASCEND and CAPACITY pivotal trials of pirfenidone were invited to participate. Following these recruitment efforts, a search of recent published literature on pirfenidone (5-year period) was conducted to gather a list of potential panelists. All panelists who actively participated (defined as completing and returning at least 2 out of 3 questionnaires including the final questionnaire and reviewing draft and final manuscripts) in our Delphi study were included as coauthors, per the International Committee of Medical Journal Editors criteria for authorship.

**Fig. 1:**
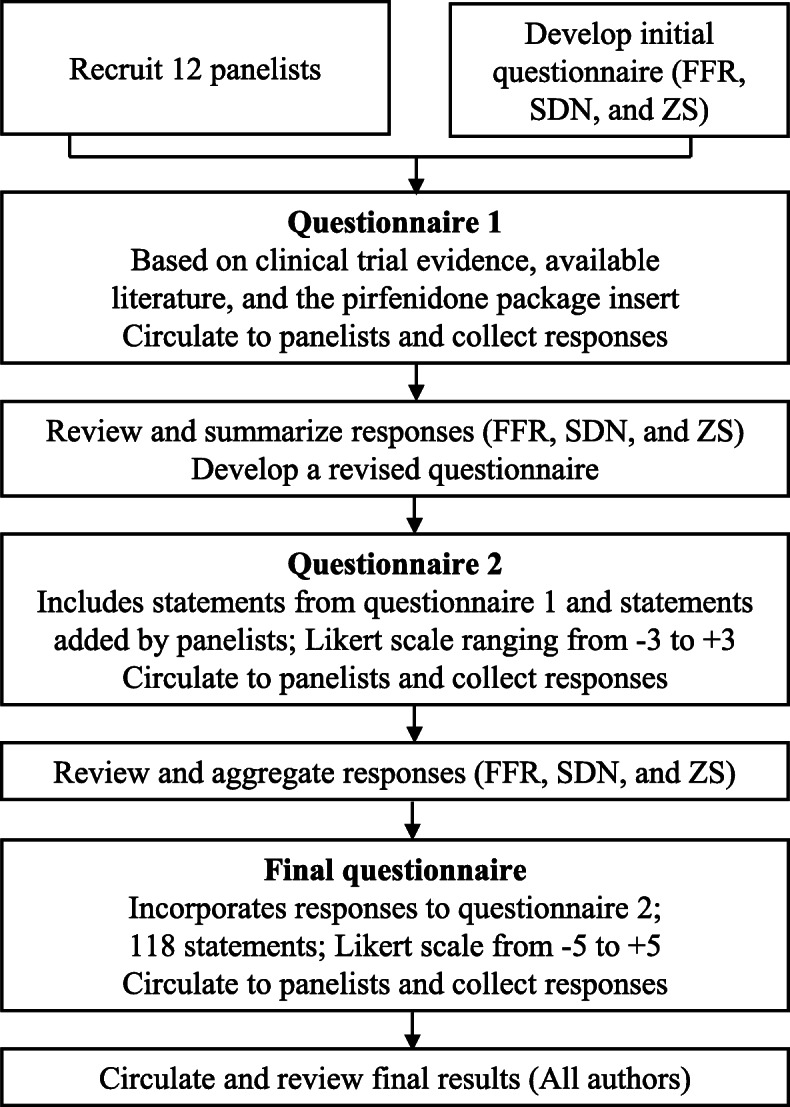
The Delphi process used in the study

Via a series of 3 questionnaires, our modified Delphi study established statements based on the prescribing information of pirfenidone, clinical trial evidence, and the panelists’ own experience and knowledge. The modified Delphi procedure has been previously described [25, 26] and key steps in the process are outlined as follows (Fig. 1):

1. Based on published literature, an initial questionnaire was developed by moderators (FFR, ZS and SDN) and distributed to the panel via an online survey platform [4, 8–10]. In this initial questionnaire panelists responded to multiple open-ended statements relevant to the use of pirfenidone, independently of one another.
2. Upon receipt of responses to the initial questionnaire, the moderators summarized the feedback and developed a second questionnaire spreadsheet that incorporated the initial statements and additional statements added by the panelists.
3. Panelists then independently voted on the second questionnaire and ranked each statement on a numeric Likert scale ranging from − 3 (strongly disagree) to + 3 (strongly agree) to establish preliminary consensus. The second questionnaire was circulated to the Delphi panelists by email and was also completed on an electronic survey platform.
4. The moderators then aggregated the responses to the second questionnaire and developed a final questionnaire as well as a feedback report for the Delphi panelists. The third and final questionnaire requested panelists to rate each statement on a numeric Likert scale ranging from − 5 (strongly disagree) to + 5 (strongly agree) to improve discriminant ability.
5. Finally, the results of the final questionnaire were aggregated into a summary spreadsheet and circulated to the panelists for review and comment.

The anonymity of the panelists was critical throughout the Delphi study, thus ensuring that no bias was induced by influential clinicians. Panelists were encouraged to provide feedback on the validity, specificity, and content of the items under consideration. All comments were incorporated verbatim and anonymity was maintained in all three rounds of the questionnaires.

**Fig. 2:**
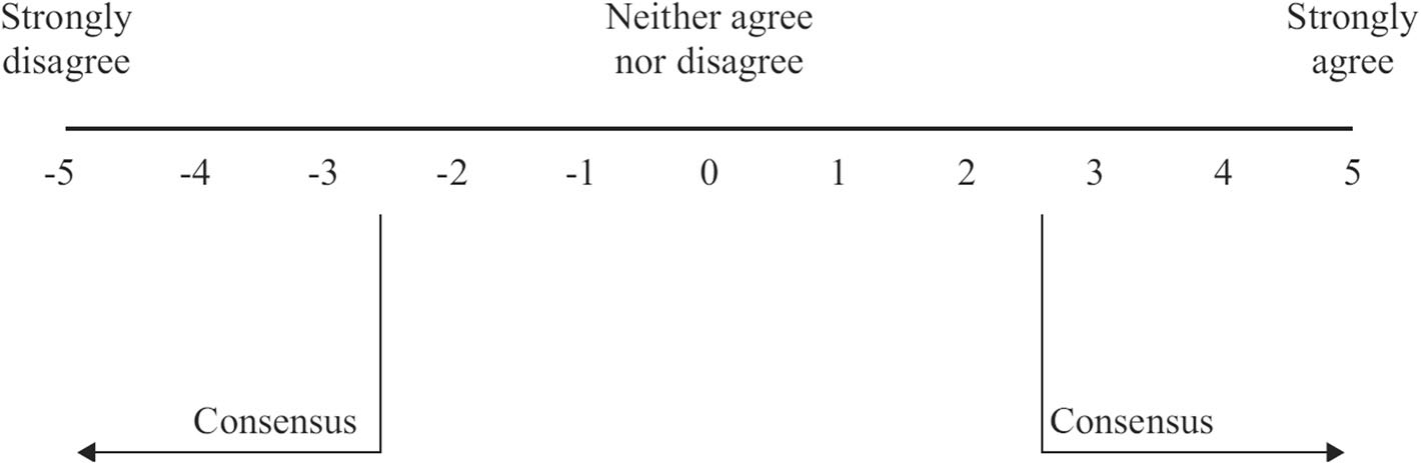
The Likert scale used in the final stage of the Delphi process. For the following Delphi Consensus Figs. 3, 4, 5, 6, 7, 8, 9, 10, statements with a consensus for are bolded; statements with consensus against are shown with a grey background

Consensus was defined when the mean rating from the panelists was ≥2.5 or ≤ − 2.5, on the Likert scale used in the final questionnaire (Fig. 2), with a standard deviation that did not cross zero [25]. Recommendations with a mean rating absolute value ≥4.0 were defined as strong. Consensus was not defined for questions answered with a time period rather than the Likert scale.

## Results

**Fig. 3:**
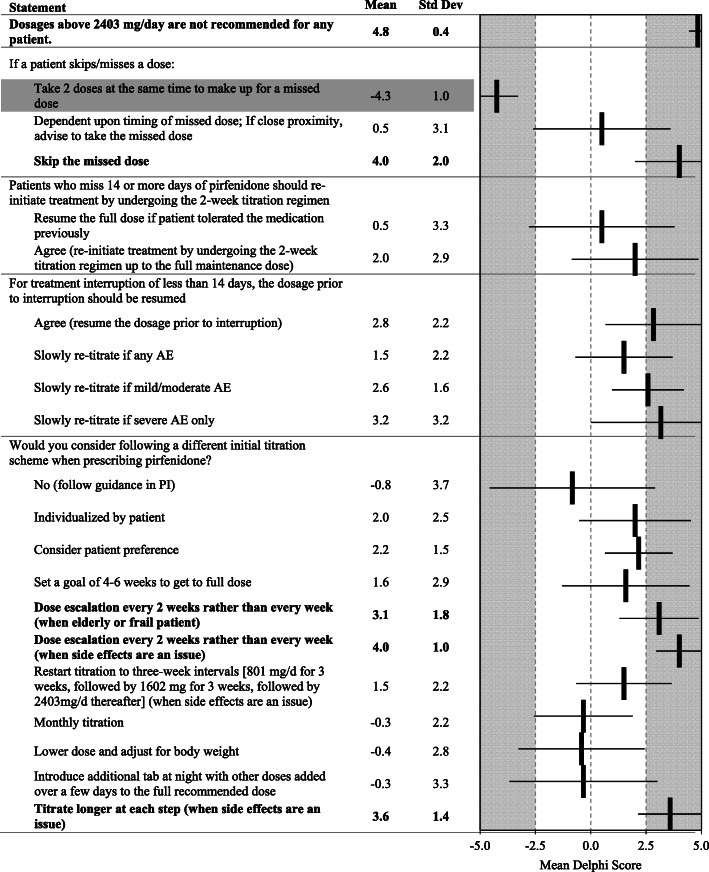
Dosing and Administration. Statements with a consensus for are bolded; statements with consensus against are shown with a grey background

**Fig. 4:**
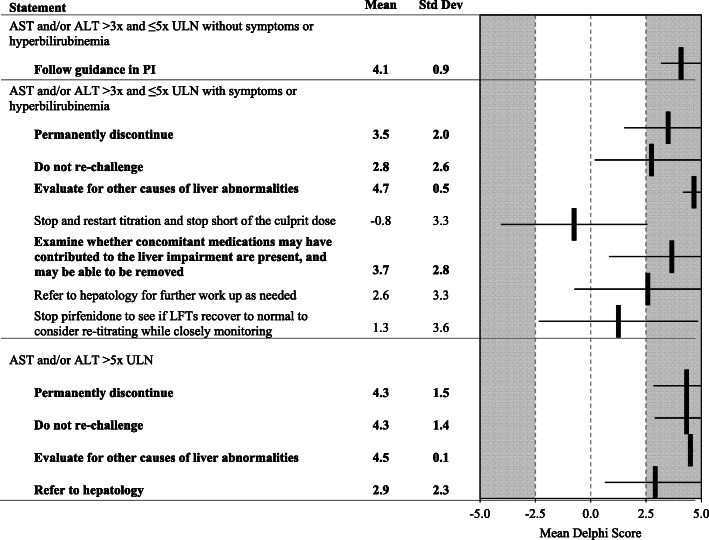
Liver Abnormalities. Statements with a consensus for are bolded; statements with consensus against are shown with a grey background

**Fig. 5:**
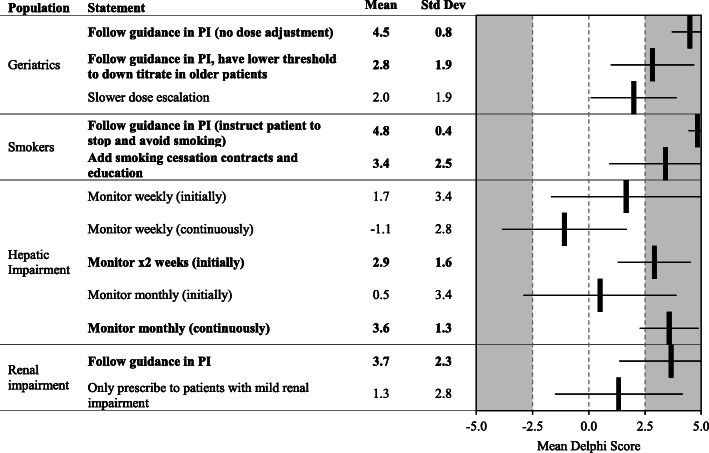
Special Populations. Statements with a consensus for are bolded; statements with consensus against are shown with a grey background

**Fig. 6:**
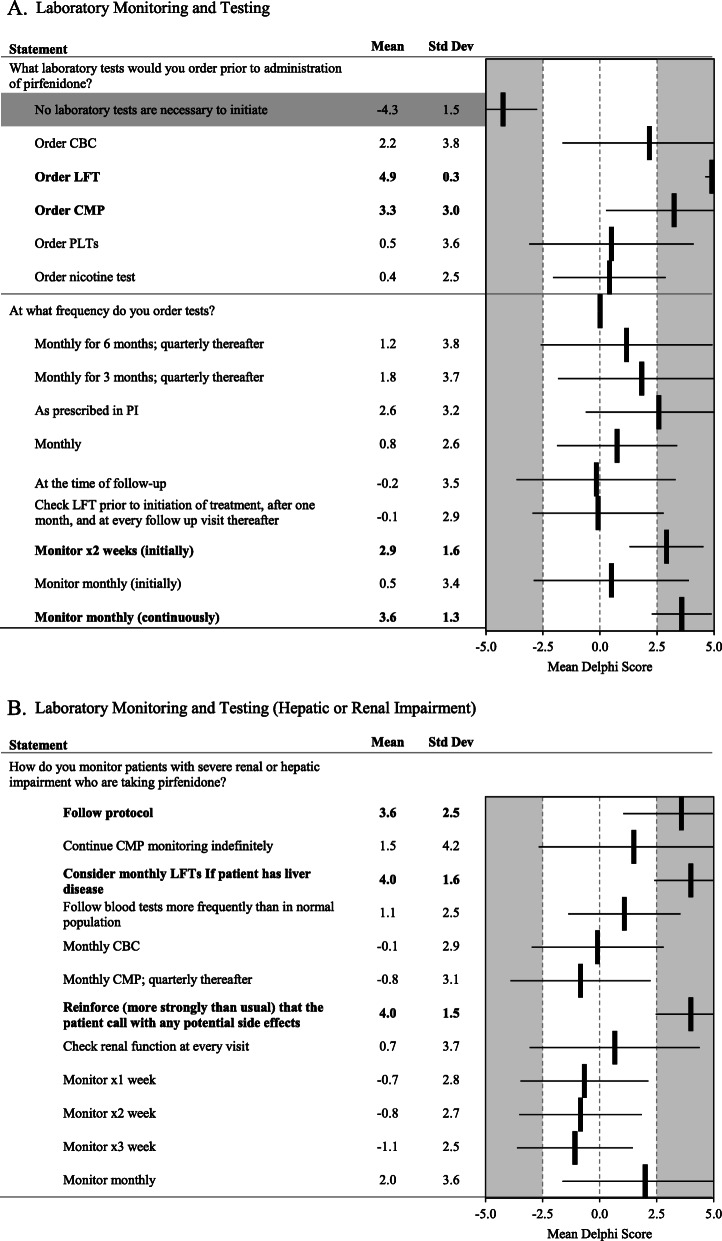
**a** Laboratory Monitoring and Testing. **b** Laboratory Monitoring and Testing (Hepatic or Renal Impairment). Statements with a consensus for are bolded; statements with consensus against are shown with a grey background

**Fig. 7:**
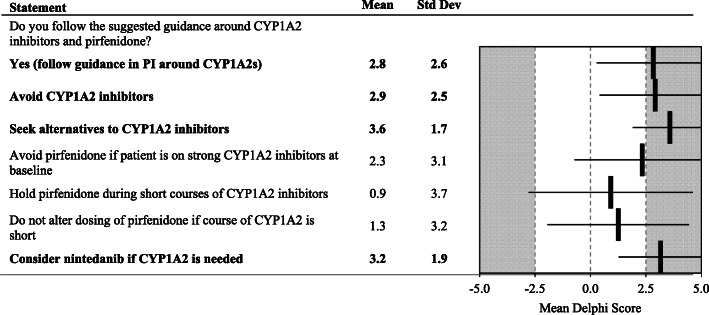
Dosing Modification due to Drug Interactions. Statements with a consensus for are bolded; statements with consensus against are shown with a grey background

**Fig. 8:**
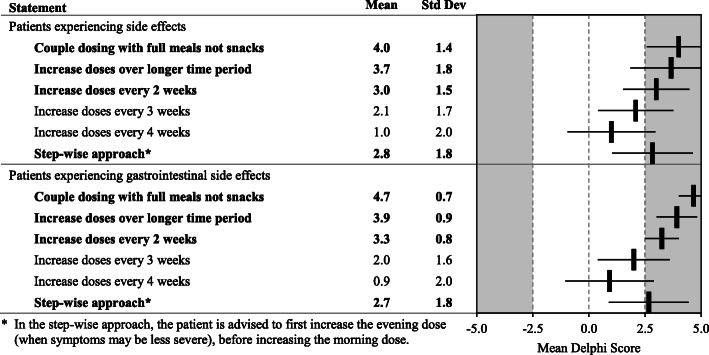
Dosing Modification due to Side Effects. Statements with a consensus for are bolded; statements with consensus against are shown with a grey background

**Fig. 9:**
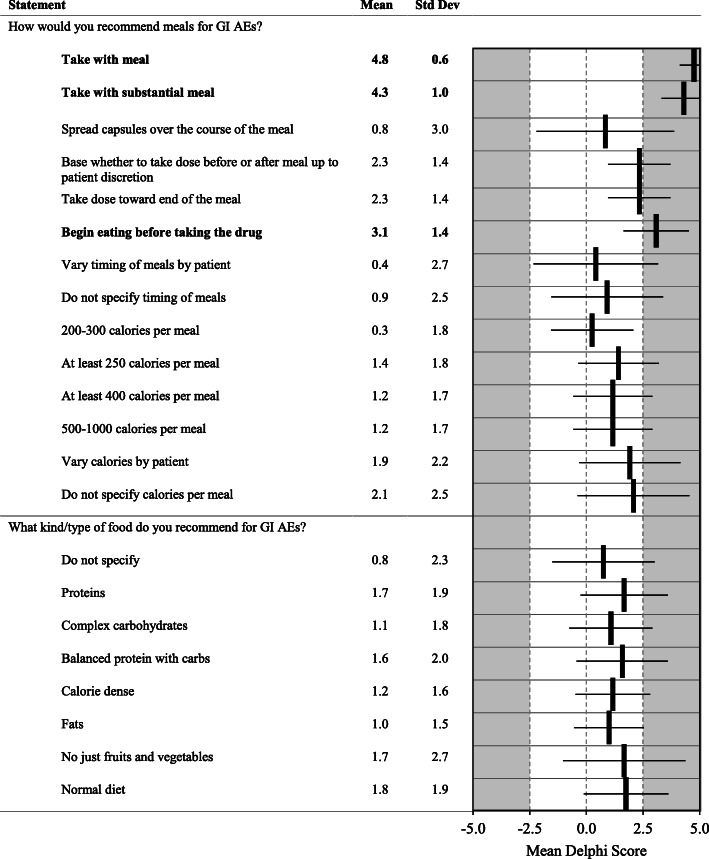
Gastrointestinal Adverse Events. Statements with a consensus for are bolded; statements with consensus against are shown with a grey background

In total, 12 physicians joined the Delphi panel, consisting of members practicing in academic (*n* = 10), hospital (*n* = 3), and Veterans Administration (*n* = 1) settings (some panelists practice across multiple care settings). Their collective experience with IPF was 17.6 ± 7.1(mean ± SD) years; treating 23.3 ± 11.1 (mean ± SD) patients with IPF per month and prescribing pirfenidone to 50.4 ± 34.6 (mean ± SD) patients. Percentages of practices devoted to patients with IPF ranged from 5 to 85% with an average of ~ 40% of their practices being devoted to treating IPF. All 12 panelists participated actively in the Delphi process. The final Delphi questionnaire was divided into 5 topics including 286 statements. All statements and the results of the final questionnaire are shown in Figs. 3, 4, 5, 6, 7, 8, 9.

Panelists strongly recommended that patients who have missed a dose of pirfenidone skip the missed dose and strongly recommended against taking two doses at the same time to make up for the missed dose. For treatment interruptions of less than 14 days, doses should be slowly re-titrated if mild/moderate or severe AEs occur. A slower titration scheme should be considered with respect to patient preference and AEs. Dose escalations can be made every 2 weeks rather than every week for elderly or frail patients or patients experiencing side effects. Panelists favorably ranked increasing doses over a longer time period (either every 2 or 3 weeks) or allowing the patient to do a step-wise approach (i.e. increase the evening dose first [when symptoms may be less] before increasing the morning dose as the optimal dosing titration scheme for patients experiencing side effects) (Fig. 3).

Panelists reached a consensus that pirfenidone should be permanently discontinued if a patient exhibits > 3 but ≤5 × upper limit of normal (ULN) alanine aminotransferase (ALT) and/or aspartate aminotransferase (AST) accompanied by symptoms or hyperbilirubinemia. These patients should not be re-challenged. Their liver abnormalities should be evaluated for other causes. Concomitant medications should be examined and evaluated for possible contribution to the liver impairment. Moreover, if a patient exhibits > 5 × ULN ALT and/or AST, pirfenidone should be permanently discontinued, the patient should not be re-challenged with pirfenidone, and their liver abnormalities should be evaluated for other causes. These patients may be referred to hepatology (Fig. 4).

The panelists strongly favored not adjusting doses for elderly populations, however, a modest consensus was obtained on having a lower threshold to down-titrate these patients, or to slow their dose escalation compared to younger subjects. Smokers should be instructed to stop smoking, and smoking cessation contracts and education should be administered. The panel came to agreement to monitor patients with hepatic impairment every 2 weeks initially then monitor monthly continuously. For patients with renal impairment, panelists strongly favored following the guidance in the package insert (Fig. 5).

The panelists were in consensus and strongly disagreed that no laboratory tests are necessary to initiate treatment with pirfenidone. They favored ordering complete blood count (CBC) and comprehensive metabolic panel (CMP). Liver function tests (LFTs) should be considered monthly, and blood tests should be followed frequently if a patient has liver disease (Fig. 6a). Reinforcement (more strongly than usual) that the patient notify the prescribing physician of any potential side effects was strongly suggested (Fig. 6b).

The group recommended following the package insert for guidance around CYP1A2 inhibitors with respect to dose modifications due to drug interactions and suggested seeking alternatives in patients being treated with pirfenidone. Nintedanib should be considered if CYP1A2 inhibitors are necessary (Fig. 7).

With specific regard to GI side effects, panelists favored coupling doses of pirfenidone with full meals, not snacks; increasing doses over longer periods of time (every 2 or 3 weeks); and allowing patients to do a step-wise approach that increases the evening dose first (when symptoms may be less) before increasing the morning dose (Fig. 8). Panelists strongly recommend patients take pirfenidone with a substantial meal. Agreement was also achieved to take the drug before eating, or toward the end of their meal, or to spread capsules over the course of the meal. No consensus was obtained on the specific make up of meals. The causes of GI AEs should be assessed for other causes beyond treatment with pirfenidone (Fig. 9).

**Fig. 10:**
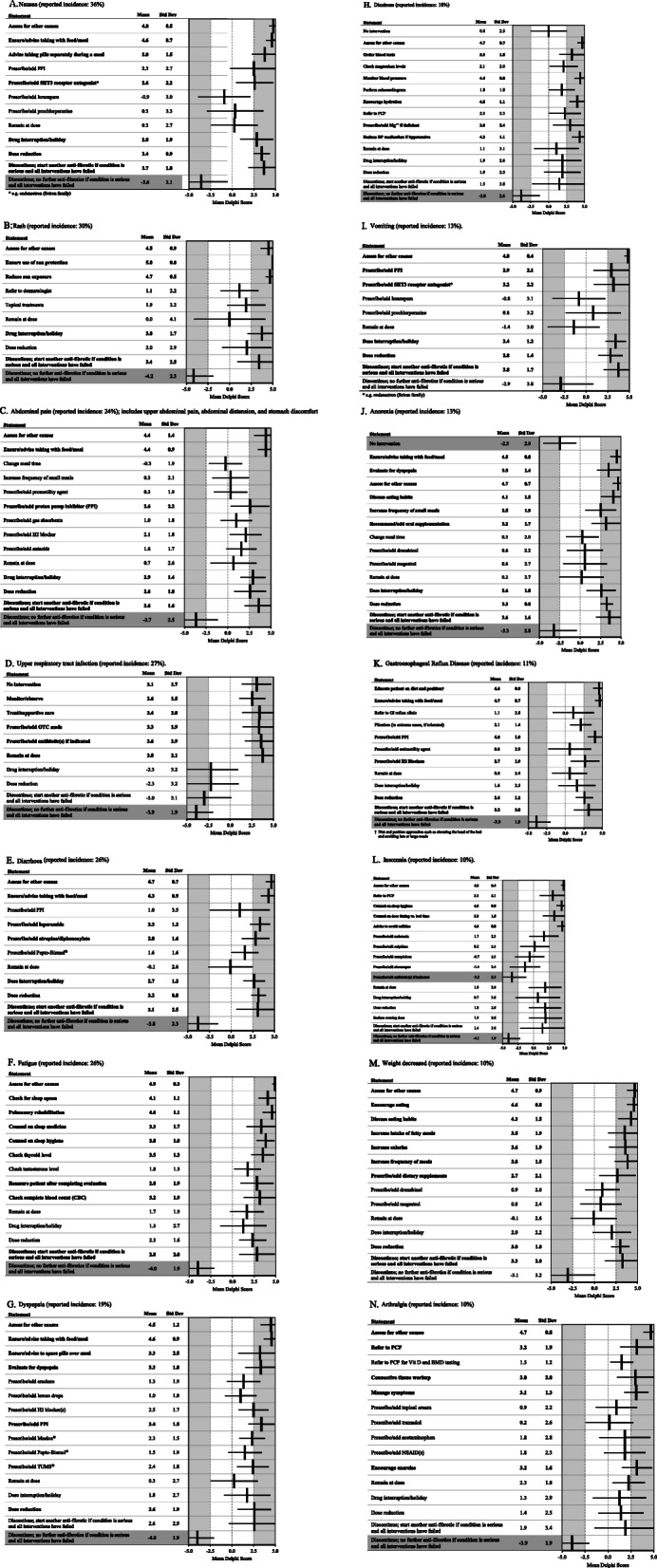
**a** Nausea (reported incidence: 36%). **b** Rash (reported incidence: 30%). **c** Abdominal pain (reported incidence: 24%); includes upper abdominal pain, abdominal distension, and stomach discomfort. **d** Upper respiratory tract infection (reported incidence: 27%). **e** Diarrhoea (reported incidence: 26%). **f** Fatigue (reported incidence: 26%). **g** Dyspepsia (reported incidence: 19%). **h** Dizziness (reported incidence: 18%). **i** Vomiting (reported incidence: 13%). **j** Anorexia (reported incidence: 13%). **k** Gastroesophageal Reflux Disease (reported incidence: 11%). **l** Insomnia (reported incidence: 10%). **m** Weight decreased (reported incidence: 10%). **n** Arthralgia (reported incidence: 10%). Statements with a consensus for are bolded; statements with consensus against are shown with a grey background

The panelists provided several consensus recommendations for management of common AEs. Figure 10a-n illustrate the strength of these recommendations.

### Rash

In a patient who experiences rash, it is important to assess for other causes of rash. Ensure the use of sun protection (e.g. protective clothing, sunscreen, long sleeves, and hats) as well as reduce the amount of sun exposure. Referral to a dermatologist is not recommended for rash. A drug interruption/holiday may be useful in these patients. Pirfenidone should be discontinued if severe, persistent rash occurs and treatment with another anti-fibrotic should be considered.

### Upper respiratory tract infections (URTI)

URTI should be monitored and treated with supportive care, over the counter (OTC) medications and/or antibiotics. Patients with URTI can remain at their prescribed dose of pirfenidone. It was not recommended to discontinue pirfenidone nor start another anti-fibrotic medication for patients with URTI.

### Dizziness

Dizziness should be evaluated for other causes. Blood tests should be ordered and levels of magnesium (Mg++) should be checked (prescribe Mg++ if deficient). Blood pressure should be monitored, and an echocardiogram can be performed. If blood pressure is low, reduce blood pressure medications. Encourage dizzy patients to stay well hydrated. It was not recommended to discontinue pirfenidone nor start another anti-fibrotic medication for patients experiencing dizziness.

### Insomnia

Assess for other causes of insomnia beyond the use of pirfenidone. Counsel the patient on sleep hygiene, timing on dose in relation to bedtime, and advise them to avoid caffeine. Referral to the patient’s primary care physician (PCP) is recommended. It was not recommended to discontinue pirfenidone nor start another anti-fibrotic medication for patients experiencing insomnia.

### Arthralgia

Arthralgia should be assessed for other causes and managed. Referral to the patient’s PCP is recommended for vitamin D level and bone density testing. A connective tissue work up should be performed prior to initializing therapy. Exercise should be encouraged. Patients experiencing arthralgia should remain at their prescribed dose of pirfenidone. It was not recommended to discontinue pirfenidone nor start another anti-fibrotic medication for patients experiencing arthralgia.

### Fatigue

Other causes of fatigue should be ruled out. Counsel patients on sleep medicine and hygiene as well as prescribe pulmonary rehab. Thyroid and testosterone levels should be checked, and a complete blood count should be performed. Patients should be reassured after they are evaluated. Dose reductions are suggested in patients with fatigue. Pirfenidone should be discontinued if severe fatigue occurs and treatment with another anti-fibrotic can be considered.

### Abdominal pain

Patients who develop abdominal pain should be ensured/advised to take their dose with food/meal. Abdominal pain should be assessed for other causes not related to pirfenidone. A PPI can be prescribed to these patients. Dose reductions and drug holidays are suggested in patients with abdominal pain. Pirfenidone should be discontinued if severe abdominal pain occurs and treatment with other anti-fibrotics can be considered.

### Anorexia

Discussing eating habits, increasing the amount of small portioned meals, and adding oral supplementation for patients experiencing anorexia is recommended. Dose reductions and drug holidays are suggested in patients with anorexia. Pirfenidone should be discontinued if after exhausting all remedies severe anorexia occurs and treatment with other anti-fibrotics can be considered.

### Diarrhea

Patients who experience diarrhea should be ensured/advised to take their dose with food/meal. Loperamide and atropine/diphenoxylate can be prescribed to these patients and dose reductions and drug holidays are suggested. After exhaustion of all remedies, pirfenidone could be discontinued if severe diarrhea occurs and treatment with another anti-fibrotic can be considered.

### Dyspepsia

Patients who experience diarrhea should be advised to take their dose with a meal and advised to spread their pills out over the course of the meal. Proton pump inhibitors (PPI) and antacids like Maalox®, and TUMS® may be prescribed. Dose reductions are recommended for patients with dyspepsia. After exhausting all remedies, pirfenidone should be discontinued if severe dyspepsia occurs and treatment with another anti-fibrotic can be considered.

### Gastro-esophageal reflux disease (GERD)

Education on diet and positional approaches, as well as ensuring that patients take their dose with meals is recommended for patients with GERD. PPIs and H2 (histamine 2) blockers should be prescribed for GERD with dose reductions and drug holidays as additional suggestions. After all remedies are exhausted, pirfenidone should be discontinued if severe GERD occurs and treatment with another anti-fibrotic can be considered.

### Nausea

Patients who experience nausea should be advised to take their dose with food and advised to take their pills separately throughout the duration of their meal. Prescribe/add 5-Hydroxytryptamine type 3 (5HT3) receptor antagonists such as ondansetron for patients with nausea, with dose reductions and drug holidays as further suggestions. After all remedies are exhausted, pirfenidone should be discontinued if severe nausea occurs and treatment with another anti-fibrotic can be considered.

### Vomiting

PPIs and 5HT3 receptor antagonists are recommended for patients who experience vomiting. Dose reductions and drug holidays are suggested and pirfenidone should be discontinued if severe vomiting occurs. In such situations, treatment with another anti-fibrotic can be considered.

### Weight loss

Encouraging eating, discussing eating habits, and increasing the frequency and size of meals are recommended for patients with decreased weight. Fatty meals are recommended as well as dietary supplements. Dose reductions are recommended for patients with decreased weight. Pirfenidone could be discontinued if after exhausting all remedies severe weight loss occurs, and treatment with another anti-fibrotic can be considered.

## Discussion

At a time where there is a lack of evidence-based real-world data, this report is intended to aid physicians who use pirfenidone in managing patients with IPF. The goal of this report is to provide expert guidance for quality treatment and care relating to pirfenidone-associated AEs and interruptions in therapy. The key to success with pirfenidone is to not only start patients on therapy early, but to maintain them on therapy as evidenced by improved mortality in treatment adherent patients [13]. With discontinuation rates of approximately 30%, any guidance that facilitates patients staying on treatment may result in improved outcomes, even at lower more tolerable doses [27]. The consensus recommendations contained within this report were developed by a panel of physicians experienced with pirfenidone and IPF using a modified Delphi methodology. The Delphi process developed a total of 286 statements and the panel reached consensus on 162 of those statements.

Our findings build on the consensus recommendations published by Costabel et al. and Lancaster et al. and further provide guidance on the most common AEs associated with pirfenidone as opposed to developing recommendations for groups of AEs as a whole [18, 19]. Strong consensus was achieved on several recommendations in our study. Panelists agreed that pirfenidone dosing titration schedules can be extended and modified when AEs or concerns for AEs are an issue. Frequent monitoring for abnormalities and coupling doses of pirfenidone with food were also suggested. Management options, including palliative medications for the most frequent AEs associated with pirfenidone are contained within. This real-world methodology on how to use and titrate pirfenidone provides very practical knowledge that augments the prescribing information and the randomized control trial methodologies to translate useful practical know-how for lesser experienced physicians.

For this study, the Delphi method was implemented using electronic communications to gather and distribute information. With this format, panelists were able to complete the surveys without stringent time restrictions or need for travel. All panelists’ opinions were weighed equally, as the Delphi process uses a systematic, anonymous process that promotes open sharing of thoughts and beliefs while making it difficult for any one panelist to dictate the process. Electronic communications were utilized so as to uphold the anonymous nature of the Delphi process.

### Limitations

The Delphi process has several limitations [25, 26], lack of a standardized consensus-defining criterion being one of the drawbacks [28–30]. Given that the study is designed to elicit guidance when no strong evidence is available, the process itself may not be statistically rigorous, and even if consensus is reached, there is no guarantee that the consensus answer is generalizable or appropriate [28, 30, 31].

Bias can inadvertently be introduced during the process of panel selection and the development of the initial questionnaire, even though there are measures in place to prevent it [23, 29]. Anonymity is an integral component of the Delphi process, but this also means lesser accountability of the panelists towards their responses, and generation of responses with insufficient or minimal consideration [28]. Panelists with relatively less experience in the field may have a voice disproportionate to their familiarity with disease treatment, as less experienced panelists can vote against and effectively limit the statistical weight of the standard methods suggested by more experienced panelists. Due to the fact that the panelists were assembled for their diverse experiences with pirfenidone and IPF, this issue is unlikely to have been significant in this study and the manuscript aims to capture the broad spectrum of opinions [23].

In this study, the selection of US-based expert panelists was limited to ≤20 participants to help make the process manageable as well as limit responses to therapeutic strategies not pertinent to the US. As a result, the Delphi consensus may not represent global perspectives. Additionally, the small sample size (*N* = 12) might indicate that certain important perspectives from a larger, more representative population of US-based physicians might have been missed in this study. By intentionally selecting a panel of clinical researchers experienced with the clinical trials for pirfenidone (as outlined in Methods), most panelists may have some association with Genentech beyond this Delphi study. Other potential stakeholders, such as patients, pharmacists, and payers, were also not included, which might have further limited the diversity of perspectives. Study parameters stated above might have introduced biases in this paper, though we hope that this is minimized by the Delphi process and study focus area which is mostly concerned about AE management and titration/use of medication and not patient selection. In spite of the small sample size, the panelists managed to achieve consensus on 57% of the statements.

## Conclusion

In conclusion, the Delphi process was used to develop expert consensus recommendations on the use of pirfenidone in managing patients with IPF to keep patients on therapy and improve outcomes. The recommendations aim to provide useful guidance on appropriate clinical management of IPF with pirfenidone, encompassing a broad experience from the real world than what has been studied in the trials.

## Data Availability

The data sets used and/or analyzed during the current study are available from the corresponding author on reasonable request.

## Abbreviations

AE: Adverse event
ALT: Alanine aminotransferase
AST: Aspartate aminotransferase
BMD: Bone mineral density
BP: Blood pressure
CBC: Complete blood count
CMP: Comprehensive metabolic panel
CYP: Cytochrome
GERD: Gastroesophageal reflux disease
GI: Gastrointestinal
H2: Histamine 2
IPF: Idiopathic pulmonary fibrosis
LFTs: Liver function tests
Mg++: Magnesium
OTC: Over the counter
PCP: Primary care physician
PI: Prescribing information
PLTs: Platelets
PPI: Proton pump inhibitor
SD: Standard deviation
ULN: Upper limit of normal
URTI: Upper respiratory tract infection
US: United States
5HT3: 5-Hydroxytryptamine type 3
